# Notes and News

**Published:** 1889-12-14

**Authors:** 


### Notes and News.

Collected and Communicated.

The Hospitals Association holds the third meeting of this session on the 18th December, when Mr. A. Wynter Blyth, medical officer of health for St. Marylebone, will deliver a lecture on "Disinfection, Hospital and Domestic." The meeting will be held in the governors' court-room of Guy's Hospital at 8 p.m., and the treasurer, Mr. E. H. Lushington, will preside. Tickets of admission may be obtained on application to the secretary, at Norfolk House, Norfolk-street, Strand.

It is noteworthy how many matrons and nurses have attended the recent monthly meetings of the Hospitals Association. We noticed, for instance, at the crowded meeting at King's College Hospital, the other evening, the following matrons amongst others: Miss K. Philippa Hicks (Hospital for Sick Children), Miss Gordon (Charing Cross Hospital), Mrs. Phillips (Queen Charlotte's Hospital), Miss E. Vincent (St. Marylebone Infirmary), Miss Ridley (Hospital for Epilepsy and Paralysis), Miss Meyrick (lady superintendent, Hospital for Gentlewomen), and Miss Coates. The meeting at Guy's Hospital on the 18th inst. is sure to be well attended, owing to the interesting and practical nature of the subject selected by Mr. Wynter Blyth, "Disinfection, Hospital and Domestic."

The Hospitals Association has completed its arrangements for the establishment and maintenance of a complete and effective street ambulance service for the metropolis, and is now awaiting the result of a trial in actual work in London of a number of ambulances of two selected patterns by the various authorities who are co-operating with the association in the movement. London has been mapped out into districts, ample accommodation for ambulance stations admirably situated has been placed at the disposal of the association, and as soon as the above trial is completed the work of ambulancing London will be rapidly proceeded with.

The quarterly meeting of governors of Carlisle Infirmary took place on Nov. 21st, the Bishop of Carlisle in the chair. Mr. Howitt was elected secretary, and Mr. Kekwich honorary surgeon dentist. Mr. Howie Boyd's resolution for increasing and strengthening the medical staff of the hospital was quashed on technical grounds. The committee, however, promised to deal with Mr. Howie Boyd's proposal without prejudice when next he brings it forward, and if they should be induced to let in the thin edge of the wedge of reform no one can tell where the good work may end.

We publish elsewhere a letter from "Spectator" on the important subject of "Professional Pressure." "Spectator" is himself a medical man in family practice, and is both successful and highly respected in his own neighbourhood. His letter will be read with much interest by many of his professional brethren.

At the meeting of the managers of the Metropolitan Asylums Board, held on the 30th ult., a report of the Ambulance Committee to the General Purposes Committee, having reference to section 6 of the Poor Law Act, 1889, was considered and its recommendations were adopted. The carriages belonging to the board will, in consequence, now be available for the conveyance of persons suffering from any dangerous infectious disorder to and from hospitals and other places, at a charge for each conveyance for the present of 5s., a discretionary power being retained to remit the charge when deemed expedient in the interest of the public health. Any of the following diseases will be regarded as dangerous infectious disorders—viz.: "smallpox, cholera, diphtheria, membranous croup, erysipelas, the disease known as scarlatina or scarlet fever, and the fevers known by any of the following names: Typhus, typhoid, enteric, relapsing, continued, or puerperal"; also measles, and such other "dangerous infectious disorders" as from time to time may require conveyance. It is stated to be desirable that a medical certificate setting forth the nature of the disease, and stating that the sufferer is fit to be removed, should be supplied to the board's officers previously to the patient's removal. Persons may, under exceptional circumstances, be conveyed to or from places outside the metropolitan area, an extra charge being made in all such cases.

Very successful was the Royal Free Hospital ball on Tuesday night at the Holborn Town Hall. A brilliant and numerous company assembled, the hall was prettily decorated with flags and banners, and dancing was kept up with spirit from 9 o'clock until 4 a.m. The chairman of the Hospital Weekly Board, Ed. Ford North, Esq., Drs. Dakin, Sidney Lloyd Smith, Collie, and Sayer, and Mr. Conrad W. Thies, the secretary, were present. About midnight dancing was interrupted for a few minutes, and Mr. North addressed the company on behalf of the institution, its officers, and its inmates, representing whom he said he had to thank all for their presence at this inaugural ball, and more particularly he wished to mention that the thanks of all connected with the institution were most heartily offered to Mr. J. Balls, to whom was due the initiative of this pleasant gathering, and to Mr. R. G. Hartley, the secretary of the ball committee, Mr. W. Brinklow, Mr. J. B. Bouyond, Mr. A. Aberg, and other gentlemen who had worked hard in his support with so excellent a result. He assured the company the thanks of all connected with the hospital were indeed offered to them for their hearty response to the efforts of the gentlemen he had named, and he expressed the hope that they might meet again on many anniversaries of this occasion. Frequent applause marked the points of Mr. North's short speech. A feature of the evening was the "Royal Free Quadrilles," specially arranged for this festival, and enthusiastically redemanded. Mr. C. Benson's quadrille band provided the music; Messrs. Beall and Co., of Holloway, the refreshments; Mr. H. R. Johnson was an able M.C.; and a gentleman from the photographic studios of Messrs. Fradelli and Young, who was in attendance, was honoured with sittings by a considerable number of the guests. As an annual event it may safely be predicted that this ball will be looked forward to with interest and pleasant anticipations in future years.

The following vacancies are announced this week, the amount of the salary in each case being given after the name of the institution:

**Resident Medical Officers.**

Loughborough Medical Aid Association. Assistant Physician—£200, house, &c.

York Dispensary. Medical Officer—£130, rooms, &c.

St. Pancras Dispensary. Medical Officer—£105, rooms, &c.

Blackburn Infirmary. Junior House Surgeon—£50, board, &c.

Doncaster Infirmary. House Surgeon—£100, board, &c.

London Throat Hospital. House Surgeon—£150, board, &c.

Worksop Dispensary. Surgeon—£110, rooms, &c.

Grantham Friendly Societies. Medical Officer—£150, rooms, &c.

Society for Promoting Christianity amongst the Jews. Medical Missionary for Safed—£200, rooms, &c.

Victoria Hospital for Children. House Surgeon—£50, board, &c.

Victoria Hospital for Children. House Physician—£50, board, &c.

Northampton Infirmary. House Surgeon—£125, board, &c.

Northampton Infirmary. Assistant House Surgeon—£80, board, &c.

**Non-Resident Medical Officers.**

Gordon Hospital for Fistula. Anæsthetist.

University of Edinburgh. Additional Examiner in Clinical Surgery—£75.

---

The Railway Officers and Servants' Association and Railway Orphan Fund is an institution established with the object of giving temporary and permanent assistance to railway servants in cases of accident or severe illness, to provide them with annuities when incapacitated for work, and at their death to assist their families. Since May, 1863, upwards of £78,000 has been distributed, and 114 annuitants have been elected to £20 each. The objects of the association are admirable, and it well merits increased support from the public.

---

Lord Balfour of Burleigh, president of the London Fever Hospital, writes to the papers to point out that the passing of the Infectious Disease (Notification) Act, 1889, which requires heads of families, occupiers of houses, and others to give information, will do little towards diminishing the prevalence of the diseases unless rigid and effective isolation is maintained. But this can rarely be done in private houses, and must rather be looked for in public institutions. For those whose position in the social scale precludes the idea of their contributing to the cost there are the hospitals of the Metropolitan Asylums Board, maintained out of the rates. On the other hand, for those who have the means to pay the full cost of their treatment and nursing ample provision is made at the London Fever Hospital, Liverpool-road, Islington, where a number of well-appointed private rooms are maintained, and are already much used by officers of the army and navy, and the professional classes generally, and their wives and families. For patients of this description the weekly charge is £3 3s., which covers the cost to which the institution is put. There is, however, a third class of persons, who, although not in precisely indigent circumstances, and anxious to contribute to the best of their ability, could ill afford the expense of a prolonged course of treatment. For such as these the hospital authorities provide in the best possible way for as long a period as may be necessary—generally from six to eight weeks—on payment of a single fee of three guineas, and even this may be remitted in whole or in part at the discretion of the committee. In making known these facts Lord Balfour of Burleigh desires also to appeal to the public for increased support.

Her Majesty the Queen has been graciously pleased to accept from the authoress a copy of the second edition of "The Management of Children," by "A Mother," published by Messrs. J. and A. Churchill.

---

The Cottage Hospital at East Grinstead is an excellent institution. Although it has not been open quite two years, it has done good work, fifty-five in-patients having been treated there. The hospital only admits the worst cases, but only four deaths are reported, and of these not one has occurred this year.

---

A meeting took place last week, at Paris, at the residence of Dr. Douglas Hogg, for the purpose of forming a centre in connection with the St. John Ambulance Association. The local committee was constituted, and consists of: President, his Excellency the Earl of Lytton; vice-presidents, Sir Richard Wallace, Dr. Loughnan, Hon. Dr. Alan Herbert, Dr. Faure Miller, and Dr. M'Gavin; chairman, Dr. Douglas Hogg; deputy chairman, Mr. A. Bowden, British Vice-Consul; committee, Rev. Howard Gill, Rev. George Washington, Messrs. Sewell, Huggons, Blackith, Dr. Hogg, sen., and Dr. Oscar Jennings. The St. John's Association is increasing rapidly in power.

---

A festival dinner in aid of the funds of the Royal Sea Bathing Infirmary, Margate, and in celebration of the hundredth anniversary of its existence, will be held at the Hotel Métropôle, on January 15th, 1890, under the presidency of Mr. Joseph Sebag Montefiore, J.P., etc., High Sheriff for Kent. The Infirmary is unique in character, being reserved exclusively for "the scrofulous poor of all England." During the century 38,816 patients have been treated there. In the year ending the 10th of July last 714 patients were received, of whom 229 were discharged as cured, 198 greatly benefited, 51 benefited, 4 died, and 38 cases were discharged as hopeless. We congratulate the infirmary on its centenary, and wish it continued prosperity.

---

The report issued by the council of the Metropolitan Hospital Sunday Fund for 1889 is eminently satisfactory. The collection this year surpassed all previous record, the total sum received being £1,100 more than on any previous occasion. £40,749 10s. 3d. was expended in awards to 104 hospitals, seven institutions, fifty dispensaries, and in surgical appliances, the total working expenses amounting to £1,478 7s. 11d. On the 11th inst. the constituents approved the report of the Committee of Distribution, and passed a cordial vote of thanks and congratulation to their president and treasurer, the Right Hon. Sir Henry Isaacs, who promises to be indefatigable in his efforts on behalf of the fund.

---

Both the president and the council deserve the congratulations of the public at large on the admirable result of their labours. We note with especial satisfaction that they have had the courage of their convictions, and have not hesitated to withhold awards when they were persuaded that the reasons for such a step were undeniably sound. The regrettable incidents at the St. John's Hospital for Diseases of the Skin, Leicester-square, led to the refusal of an award, and few people will decline to endorse the action of the council in this respect. Altogether, the seventeenth year of the fund's existence has been a bright one, and should be the precursor of still greater triumphs in the future.

